# SiMul-db: a database of single and multi-target Cas9 guides for hazelnut editing

**DOI:** 10.3389/fgene.2024.1467316

**Published:** 2024-12-16

**Authors:** Ciro Gianmaria Amoroso, Giuseppe Andolfo

**Affiliations:** Department of Agricultural Sciences, University of Naples ‘Federico II’, Portici, Italy

**Keywords:** gRNA design, Corylus sp., paralogs, orthology analysis, gene editing

## 1 Introduction

### 1.1 Hazelnut cultivation and challenges

Hazelnuts are trees belonging to the Betulaceae family and *Corylus* genus (Wani et al., 2020). Due to their delicious flavor profile, nutrient composition, and antioxidant properties, hazelnuts are widely used as whole nuts or as processed foods. Due to their highly appreciated properties, popular *Corylus* species are cultivated across the globe, including *Corylus avellana*, widely cultivated in Europe; *C. americana*, predominantly found in North America; and *C. heterophylla* and *C. mandshurica*, extensively utilized in Asia (Botta et al., 2019). Thus far, several pathogens such as *Xanthomonas* sp., *Pseudomonas* sp., *Botrytis cinerea*, *Alternaria* sp., *Cytospora* sp. *Phytophthora* sp. and various pests compromise nut production (Guerrero et al., 2014; Battilani et al., 2018; Sun et al., 2023), which also constantly faces environmental stress (Allegrini et al., 2022).

### 1.2 Limitations of classical breeding and the potential of CRISPR-Cas9

Plants are constantly engaged in a struggle for survival and adaptation, conventional breeding techniques have allowed the development of hazelnut cultivars with improved characteristics especially related to cold resistance and yield (Wang G. X. et al., 2018; Botta et al., 2019; Mehlenbacher and Molnar, 2021). However, the efficiency of classical breeding approaches depends on the availability of genomic resources and may be limited in commercial varieties due to the introduction of undesired genetic traits during breeding steps. Furthermore, the classical breeding approach has been recognized as a time-consuming process that requires multiple generations and years to introduce and fix desirable traits (Tester and Langridge, 2010). Significant support for hazelnut genetic research and improvement may come from new genome editing techniques that are revolutionizing plant breeding programs and functional studies. In particular, the Clustered Regularly Interspaced Short Palindromic Repeats (CRISPR)-Cas9 technology has been successfully applied in various fruit trees and nuts, becoming a conventional technique for enhancing biotic and abiotic stress tolerance in plants (Wang X. et al., 2018; Chang et al., 2022). The CRISPR-Cas9 system employs the Cas9 nuclease, able to induce DNA double-strand breaks (DSB) (Puchta, 2017). Once a DSB is created, the cell’s natural repair mechanisms come into play, leading to non-homologous end joining (NHEJ) or homology-directed repair (HDR). NHEJ often leads to insertions or deletions (indels) at the break site, which can result in gene knockout, while HDR can be utilized for precise edits when a donor template is provided (Khan et al., 2018). The guidance for these modifications is provided by guide RNAs (gRNAs) that are designed to effectively guide the Cas9 nuclease on the intended target sites (Hsu et al., 2013). The success of these modifications heavily relies on the design of highly specific gRNAs (Filippova et al., 2019). For example (Evangelista et al., 2024), suggested that the design of gRNAs targeting specific domains of hazelnut allergenic genes could reduce unintended effects caused by complete gene silencing. This approach would enhance the hypo-allergenicity of plants without compromising gene fitness (Tran et al., 2021). Indeed, this strategy has been employed in previous studies where the Cas9 enzyme was directed toward specific domains associated with plant stress susceptibility (Tran et al., 2021). Thereby, the CRISPR-Cas9 technology has been defined as a simple, highly efficient, specific, and cost-effective method that can facilitate functional genetic studies and the generation of transgene-free edited plants in a shorter period compared to classical breeding.

### 1.3 gRNA design for CRISPR-Cas9 in hazelnut

Numerous web-based tools have been developed to facilitate the design of gRNAs across a variety of plant species. Available online software can be used for selecting optimal sgRNA targets based on user-defined parameters (Uniyal et al., 2019; Kornel et al., 2019; Haeussler et al., 2016; Liu et al., 2017; Bae et al., 2014a). However, to date, user-friendly software integrating the genomes of *Corylus* species are not yet available, which presents a significant gap for researchers in this area. Crucial support for the application of CRISPR-Cas9 in hazelnut came from recent studies that released genome assemblies of different *Corylus* species, providing insights into genetic diversity and evolutionary gene relationships (Li et al., 2021; Lucas et al., 2021; Zhao et al., 2021; Brainard et al., 2024). High-quality genome sequences and curated gene prediction are essential for identifying suitable targets and gRNA design (Mohr et al., 2016). However, several factors influence the gRNA effectiveness, efficiency, and uniqueness of target genes, such as the sequences matching on the target gene, the position of the Protospacer Adjacent Motif (PAM) sequence, the accessibility of target sites within the chromatin structure (Jensen et al., 2017), and the formation of secondary structures (Riesenberg et al., 2022). Indeed, it has been shown that self-folding free energy strongly influences cleavage efficiency (Wang et al., 2019). Therefore, gRNA activity is predicted by specific methods providing on and off-target scores for evaluating the potential cutting efficiency of gRNAs on target genes and on potential unintended genomic loci (Mohr et al., 2016). However, currently available tools for gRNA design do not allow for determining their secondary structure (Hassan et al., 2021). Guides are predicted by assessing their activity through various tools that have been developed (Bae et al., 2014b; Montague et al., 2014; Moreno-Mateos et al., 2015; Chuai et al., 2018; Concordet and Haeussler, 2018; Cui et al., 2018). Algorithms such as Rule Set one and Rule Set 2 have been developed for on-target activity prediction (Gagnon et al., 2014; Heigwer et al., 2014; Wang et al., 2014; Xu et al., 2015). These algorithms take into account features like nucleotide composition, GC content, and positional characteristics to forecast gRNA efficacy with the objective of enhancing gRNA design by maximizing on-target activity. Conversely, for predicting off-target effects, algorithms like CFD (Cutting Frequency Determination), Mismatch count, and MIT specificity have been developed (Cong et al., 2013; Hsu et al., 2013; Mali et al., 2013; Doench et al., 2014; Doench et al., 2016). These algorithms employ scoring systems based on mismatches and sequence features to anticipate potential off-target activity of gRNAs. Recent studies pointed out the reliability and accuracy of the CFD score compared to the MIT score and Mismatch Count method in predicting off-target effects during gRNA design for CRISPR-Cas9 applications in plants (Liu et al., 2020; Naeem et al., 2020). The development of dedicated databases (DB) is a real support for molecular biologists in genome editing programs. Available user-friendly tools lacked *Corylus* reference genomes, and bioinformatics software for custom analysis requires advanced command-line skills. This limitation made it difficult for researchers to access simple and intuitive interfaces for designing gRNAs. Additionally, gene editing studies require the identification of duplicated target genes (paralogs). Plant genomes frequently host gene groups that have evolved from a common ancestor retaining overlapping or redundant functions. This poses a challenge to functional genetics research and makes gRNA design a crucial step (Bhuyan et al., 2023). Therefore, an atlas could support the selection of gRNAs for the simultaneous silencing of duplicated genes, or for utilizing of Homologs Direct Repair approaches (Aksoy et al., 2022). In this view, the development of a comprehensive DB containing all this information represents a significant advantage for one of the most critical steps in CRISPR-Cas9 application.

To this end, we released the single and multi-target Cas9 guide database (SiMul-db) including gRNAs libraries, guide self-folding free energy, paralog gene lists, and protein domain annotations for *C. americana*, *C. avellana*, *C. heterophylla,* and *C. mandshurica*. Moreover, we included *Arabidopsis thaliana* in the orthology analysis for comparative proposes. Finally, we reported two examples of guide identification for singular and multiple editing of *B. cinerea* susceptible genes in *C. avellana*.

## 2 Value of the data

• SiMul-db represents a valuable genomic resource for scientists involved in hazelnut breeding programs.
• Paralog identification will facilitate the selection of gRNA for multi-copy gene targets.
• Orthology inference will permit the transfer of gene function from model species to *Corylus* genes.

## 3 Materials and methods

### 3.1 Data sources

To develop a comprehensive and user-friendly database of Cas9 guide sequences for hazelnut plants, we used the European hazelnut (*C. avellana*) ‘Tombul’ genome (v2.4) and its gene model annotation as reported by Lucas et al. (2021). The *C. avellana* genome sequence (GCA_901000735.2_CavTom2PMs-1.0_genomic.fna), gene model annotation (GCA_901000735.2_CavTom2PMs-1.0_genomic.gbff) and relative protein sequences were downloaded from the GenBank site (https://ftp.ncbi.nih.gov/genomes/genbank/plant/). We also used a genome assembly (Camericanavar_rush_835_v1.0.fa) of the American hazelnut (*C. americana*) accession ‘Rush’ (Brainard et al., 2024) and the genome assemblies (Chr_genome_assembly_changed.fa and Cma.genome.chr.fa) of two wild Asian varieties (*C. heterophylla* Fisch. and *C. mandshurica* Maxim.) (Zhao et al., 2021; Li et al., 2021), as well as *A. thaliana* genome assembly (Araport11) for comparative purposes (https://www.arabidopsis.org/).

### 3.2 CRISPR-Cas9 guide RNA design

To obtain the *Corylus* whole-genome gRNA libraries we used the reference gRNAs database (RD)-build model implemented in CRISPR-Local software using -U 15 -D 3 settings (Sun et al., 2019). The reference genomes (.fa) and corresponding gene annotations (.gff) of *C. avellana, C. americana, C. heterophylla,* and *C. mandshurica* were used as input files. The screening of all possible on-target gRNAs and their scoring were based on the Rule Set 2 algorithm (Doench et al., 2016). While the prediction of the effects of each off-target site with the highest cutting frequency determination (CFD) score for each gRNA, was realized by the SeqMap program (Jiang and Wong, 2008). All target and off-target data determined across the entire genome are exported into RD format (Supplementary Tables S1–S4), which includes information about guide sequence, physical position, the relative position against transcription start site, on-target score, and potential off-target sites with the highest CFD score for each gRNA for every locus. Database (DB)-search model was used to obtain sorted results from all annotated Cor a genes. Paralogs (PL)-search model was used to extract gRNAs matching multi-gene targets.

### 3.3 Orthology relationships, paralog genes identification and protein domains annotation

To provide a deep understanding of the evolution and diversification of genes in *Corylus* plants, we used OrthoFinder v2.5.1 package tools (Emms and Kelly, 2019). Simultaneously, *C. americana*, *C. avellana C. heterophylla*, *C. mandshurica,* and *A. thaliana* proteomes were analyzed, with default settings. In this package, the BLAST tool was used for fast sequence similarity searches among protein sequences. The clustering of genes was inferred using the MCL clustering algorithm; an unrooted gene tree was inferred for each orthogroup using DendroBLAST (Kelly and Maini, 2013). The protein domain architecture was annotated using Pfam database implemented in InterProScan v5.69–101.0 software (Jones et al., 2014) with default setting.

### 3.4 Prediction of RNAs secondary structure

The RNA secondary structure prediction and comparison were calculated with RNAfold software implemented in the ViennaRNA package (version 2.6.4) (Lorenz et al., 2011). Specifically, the propensity to form secondary structures was determined by calculating the self-folding free energy (ΔG expressed in kcal/mol) of the guide sequence using the -d2 option as the default dangling-end model, allowing a single nucleotide to contribute with all its possible favorable interactions.

## 4 Results and discussion

### 4.1 Cas9 gRNA sequences and orthogroups identification

Over thirteen million gRNAs were predicted in the four *Corylus* genome assemblies available to date (Table 1). Future updates to SiMul-db will incorporate newly sequenced *Corylus* genome assemblies, further expanding the database and increasing the number of available species for gRNA design. The guide on-target values range from 0 to 1, and gRNAs with higher on-target scores are considered to perform better (Bae et al., 2014a). Considering the high number of obtained gRNAs, we selected gRNAs with on-target score higher than 0.66, obtaining a subset of 1,025,628 gRNAs that were considered top rank (Doench et al., 2016; Haeussler et al., 2016). Interestingly, 71,262 gRNAs were classified as multi-target gRNAs (Table 1). On average, non-functional guide sequences had significantly higher potential for self-folding than functional ones (Wong et al., 2015). To hone gRNA evaluation, we estimated the self-folding free energy (ΔG) to determine guide propensity to form secondary structures. (Wong et al., 2015). Generally, gRNA will fold within itself when the ΔG value is more negative, which hinders pairing with the on-target (KesavanNair, 2023). According to Jensen et al. (2017), the ability of Cas9 endonuclease to efficiently cleave the target is greater for ΔG values comprised between −2 and 0 kcal/mol. In our database, ∼80% of best gRNAs showed a ΔG > −2 kcal/mol. Furthermore, our dataset was implemented with an orthology analysis between *Corylus* proteomes, and including *A. thaliana* as an outgroup genome. *A. thaliana* was chosen as a reference due to its widespread use as a model species and the extensive knowledge about its genes (Cao et al., 2011). Orthology analysis allowed the identification of 21,237 orthogroups (Supplementary Tables S5, S6; Supplementary Figure S1). The identification of orthogroups between *Corylus* spp. and *A. thaliana* can speed up the discovery of target genes and potential paralogs for future genome editing studies (Mota et al., 2020). Moreover, SiMul-db was implemented with gene domain predictions that could allow the selection of specific gRNAs tailored on domain of interests (Supplementary Tables S7–S10). Finally, we selected the best gRNAs with higher on-target scores and lower CFD scores for each gene model predicted in the four *Corylus* genomes (Table 1).

**TABLE 1:** Genome editing gRNA libraries for four hazelnut species.

Plant species	Gene locus n	Predicted gRNA n	Best gRNA n^a^	Multi-target gRNA n^a^
*Corylus americana*	24.562	2.949.056	233.106	15.477
*Corylus avellana*	27.271	3.737.705	288.360	26.910
*Corylus heterophylla*	27.591	3.426.099	268.341	14.640
*Corylus mandshurica*	28.409	2.948.253	235.821	14.235
**Total**	**107.833**	**13.061.113**	**1.025.628**	**71.262**

^a^ CRISPR-Cas9 gRNAs, were filtered for on-target score >0.66 and lowest CFD, score.

### 4.2 Framework of SiMul database

**FIGURE 1:**
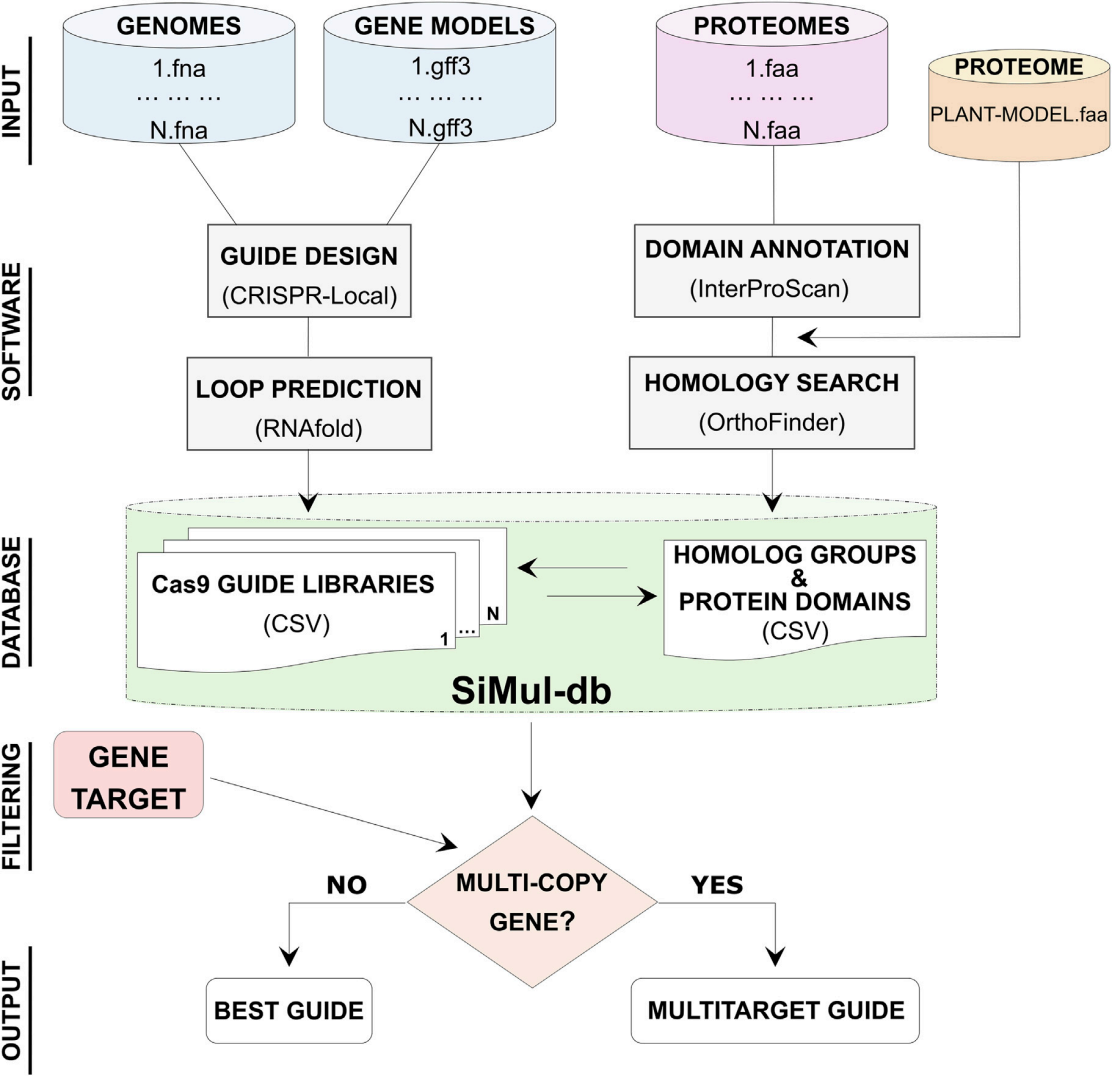
Diagram showing the workflow steps (data elaboration and primary curation) for the development of single and multi-target Cas9 gRNA database (SiMul-db). Best guide refers to gRNA with I) the top on-target, CFD and ΔG scores, or II) more suitable protein-coding region.

SiMul-db is a user-friendly research tool for the selection of the best Cas9 guides in hazelnut species. *Corylus* genomic data, including genome sequences, gene models, and protein sequences were processed to generate SiMul-db (Figure 1). Protein domain information have been obtainted consulting the Protein family database (Pfam) implemented in InterProScan software (Jones et al., 2014). Additionally, the proteome of reference model *A. thaliana* was included in orthology analysis for comparative purposes. Guide prediction and comparative analysis allowed to provide Cas9-gRNAs libraries and to identify homolog groups, respectively (Figure 1). While ΔG estimation provided additional information for a more accurate selection of guides. Therefore, through SiMul-db workflow users can identify single or multi-target genes (Armario Najera et al., 2019). Users can choose the best gRNAs considering the efficiency (on target, CFD, and ΔG) scores, or specific target region of the coding sequence, such as specific predicted domains (Supplementary Tables S7–S10). While for duplicated genes SiMul-db suggests common gRNA sequences for multi-editing (Figure 1). This streamlined approach allows for efficient and accurate guide selection for biotechnology assisted breeding in the *Corylus* genus.

### 4.3 Filtering of single and multi-targeting gRNAs

Specific hazelnut genes related to agricultural traits, metabolic pathways, or responses to biotic and abiotic stresses could be selected using SiMul-db. For example, nuts are vulnerable to *B. cinerea*, commonly known as “gray mold”, a fungal pathogen affecting various plant species (Romanazzi and Feliziani, 2014). This pathogen can infect multiple parts of hazelnut, including fruits, inducing significant yield losses and quality deterioration (Guerrero et al., 2014). SiMul-db can assist in identifying potential genes and provide valuable insights for the success of genome editing strategies. Below we provide two strategies for the selection of single and multiple gRNAs for targeting genes involved in *B. cinerea* interaction. Previous studies allowed the identification of two genes, *AtDND1* (AT5G15410) and *AtPUB17* (AT1G29340), potentially involved in plant-pathogen susceptibility (Sun et al., 2017; Ramirez Gaona et al., 2023). In particular, the silencing of *AtDND1* and *AtPUB17* has been shown to reduce susceptibility to *B. cinerea* (Supplementary Table S7). Therefore, exploring SiMul-db, researchers can easily reveal the *Corylus* orthologs (OG0011955: CamerRush.05G196000.1, Cav05g20890.1, EVM0018229.1, and CmaG0015144.1) to *AtDND1* (Supplementary Table S5), and find the best guide for each identified orthologous gene (Supplementary Table S11). Furthermore, three paralogs to *AtPUB17* were found in *C. avellana* (Cav02g18830.1, Cav02g18860.1, Cav02g18960.1). By querying SiMul-db, it was possible to identify a single gRNA that could be used for silencing all three paralogs simultaneously (Supplementary Table S12).

## 5 Conclusion

SiMul-db emerges as an innovative tool for accelerating gRNA selection for genome editing in hazelnuts. It provides lists of gRNAs with high on-target efficiency, low off-target effects, and relative self-folding free energy of the guide sequences. For the first time, the evolutionary relationships of *Cor*ylus spp. are consolidated into a unique database, which reduces the risk of undesired off-target effects and enhances the accuracy of CRISPR-Cas9. Even in the absence of efficient agrobacterium-mediated transformation protocols, SiMul-db can be consulted with alternative transformation methods, such as transient CRISPR-Cas9 modifications (Son and Park, 2022). Furthermore, future implementations of SiMul-db will include other plant species, making genome editing more accessible to researchers. This will facilitate plant genome editing programs and functional studies, ultimately boosting agricultural productivity and plant resilience.

## 6 Direct link to deposited data and information to users

The CRISPR-Cas9 gRNA dataset of four *Corylus* species can be accessed at FIGSHARE with the following link https://figshare.com/s/3ac61758f15226572aef. The candidate gRNAs identified from CRISPR-Local could be exported in GFF format and imported into the IGV genome browser (Thorvaldsdóttir et al., 2013) for comparison and visual inspection (Supplementary Figure S1). The Supplementary Material (Supplementary Tables S1–S8) for this article can be found online at: https://figshare.com/s/3ac61758f15226572aef. Users can download and use the data freely for research purpose only with acknowledgment to us and quoting this paper as a reference to the data.

## Data Availability

The original contributions presented in the study are publicly available. This data can be found here: https://figshare.com/s/3ac61758f15226572aef.

## Supplementary material

SUPPLEMENTARY FIGURE S1 A typical window in the Integrative Genomics Viewer (IGV) software. Displayed are the gene model Cav01g18280.1 located on chromosome 1 of the *C. avellana* genome and the gRNA predicted by CRISPR-Local.
