# Supplementary figures and images for: SiMul-db: a database of single and multi-target Cas9 guides for hazelnut editing
Source: Front Genet. 2024 Dec 16;15:1467316. doi: 10.3389/fgene.2024.1467316 (PMC11683083; doi:10.3389/fgene.2024.1467316)

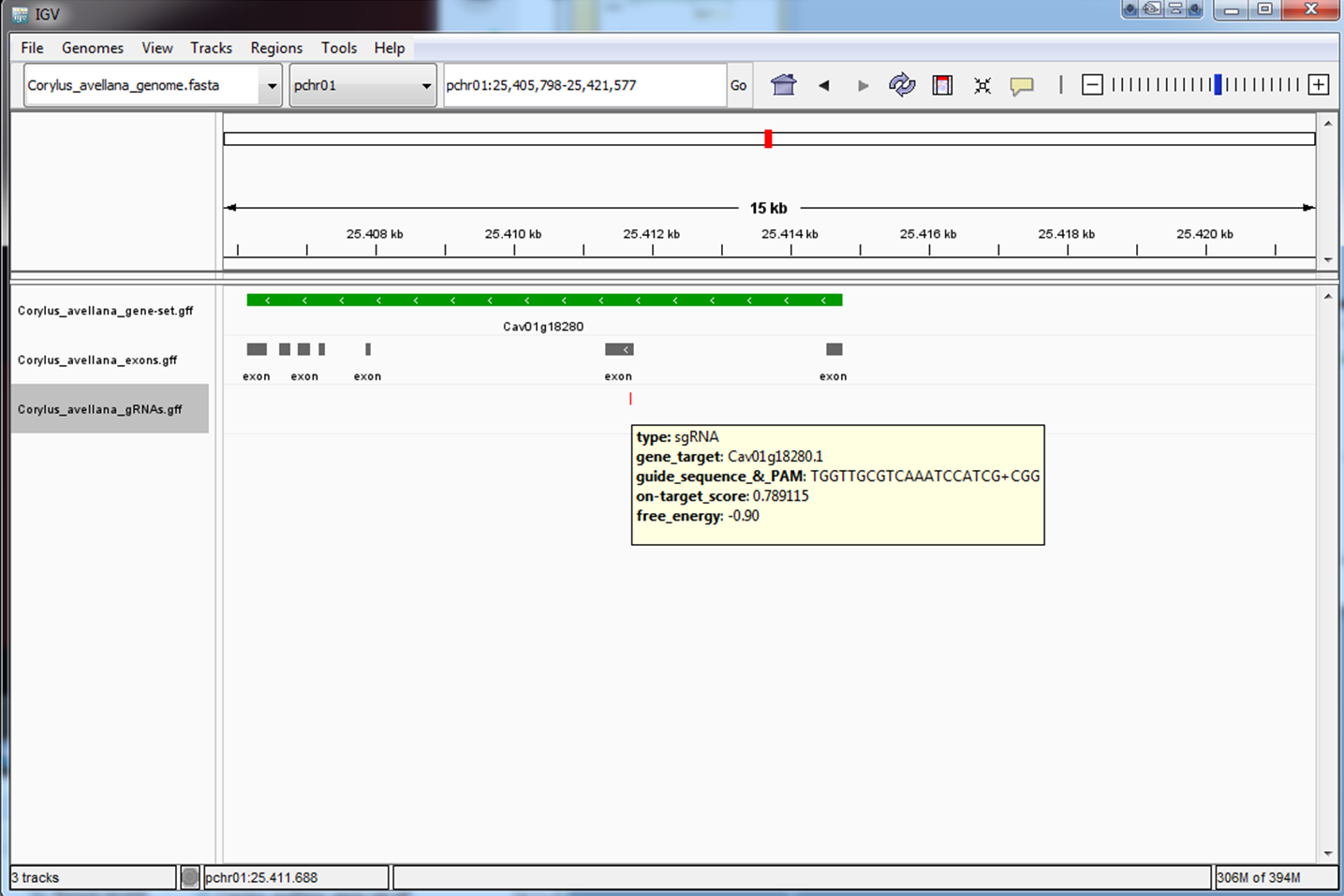

Supplement: Supplementary file 1 [file Image1.tif]
